# Comparative chromosome mapping of repetitive sequences. Implications for genomic evolution in the fish, *Hoplias malabaricus*

**DOI:** 10.1186/1471-2156-10-34

**Published:** 2009-07-07

**Authors:** Marcelo B Cioffi, Cesar Martins, Luiz AC Bertollo

**Affiliations:** 1 Universidade Federal de São Carlos, Departamento de Genética e Evolução, São Carlos, SP, Brazil; 2 UNESP – Universidade Estadual Paulista, Instituto de Biociências, Departamento de Morfologia, Botucatu, SP, Brazil

## Abstract

**Background:**

Seven karyomorphs of the fish, *Hoplias malabaricus *(A-G) were previously included in two major groups, Group I (A, B, C, D) and Group II (E, F, G), based on their similar karyotype structure. In this paper, karyomorphs from Group I were analyzed by means of distinct chromosomal markers, including silver-stained nucleolar organizer regions (Ag-NORs) and chromosomal location of repetitive sequences (18S and 5S rDNA, and satellite 5S*Hind*III-DNA), through fluorescence in situ hybridization (FISH), in order to evaluate the evolutionary relationships among them.

**Results:**

The results showed that several chromosomal markers had conserved location in the four karyomorphs. In addition, some other markers were only conserved in corresponding chromosomes of karyomorphs A-B and C-D. These data therefore reinforced and confirmed the proposed grouping of karyomorphs A-D in Group I and highlight a closer relationship between karyomorphs A-B and C-D. Moreover, the mapping pattern of some markers on some autosomes and on the chromosomes of the XY and X_1_X_2_Y systems provided new evidence concerning the possible origin of the sex chromosomes.

**Conclusion:**

The *in situ *investigation of repetitive DNA sequences adds new informative characters useful in comparative genomics at chromosomal level and provides insights into the evolutionary relationships among *Hoplias malabaricus *karyomorphs.

## Background

Although usually reported as a single taxonomic entity, *Hoplias malabaricus *(Characiformes, Erythrinidae) has significant karyotypic diversity and well-defined population differences concerning the diploid number, morphology of chromosomes and sex chromosome systems. Such intraspecific diversity enabled the characterization of seven main karyomorphs (A-G), in which those without differentiated sex chromosome systems (A, C, E and F) show a wider geographical distribution compared to those that have such systems (B, D and G), which highlights the derivative character of the latter [1]. Despite differences in diploid chromosome number and in the presence or absence of differentiated sex chromosome systems, the seven karyomorphs were subdivided into two major groups (I and II) based on general karyotype similarity [1]. Thus, Group I included karyomorphs A-D, while Group II included karyomorphs E-G (Table 1).

**Table 1:** Karyomorphs previously identified in *Hoplias malabaricus *according to Bertollo et al. (2000).

Karyomorphs	Chromosome number	Sex Chromosomes	Geographic occurrence
**Group I: first chromosome pairs of similar sizes**			
Karyomorph A	2n = 42	-	Northen to southern Brazil, Uruguay and northen Argentina
Karyomorph B	2n = 42	XX/XY	Vale do Rio Doce (Minas Gerais State) and Iguaçú River (Paraná State, Brazil)
Karyomorph C	2n = 40	-	Northen Brazil to northen Argentina
Karyomorph D	Female - 2n = 40/Male - 2n = 39	X_1_X_1_X_2_X_2_/X_1_X_2_Y	Upper Paraná hydrographic basin, Brazil
**Group II: first chromosome pairs with clearly distinct sizes**			
Karyomorph E	2n = 42	-	Trombetas River (Paraná State, Brazil)
Karyomorph F	2n = 40	-	Surinam to southeastern Brazil
Karyomorph G	Female - 2n = 40/Male - 2n = 41	XX/XY_1_Y_2_	Amazonian Rivers, Brazil

The karyotype diversity in *H. malabaricus *indicates the probable occurrence of distinct species, which is reinforced by the sympatry between some karyomorphs, without evidence of gene flow between them [1]. Specifically for karyomorphs A and C, and for karyomorphs A and D, the results obtained using RAPD-PCR genomic markers were also consistent with a lack of gene flow, providing additional evidence for karyomorphs as distinct evolutionary units [2].

Among Erythrinidae, a repetitive DNA class named 5S*Hind*III-DNA that shares similarities to 5S rDNA "true" repeats was previously isolated and characterized [3]. This sequence could not be found in the chromosomes of *Erythrinus*, *Hoplerythrinus *or *Hoplias lacerdae *and is therefore likely to be exclusive to *H. malabaricus*. Its exclusive presence in this species shows that this repetitive DNA class probably originated after the divergence of the three Erythrinidae genera and *Hoplias *species [4]. Ribosomal genes (18S and 5S rDNA) were also useful markers in *H. malabaricus*, showing significant differences among populations of this species [5,6].

Molecular organization and cytogenetic mapping of ribosomal genes and other repetitive DNA sequences have provided important contributions to the characterization of biodiversity and the evolution of ichthyofauna [7-9]. In fact, a substantial fraction of any eukaryotic genome consists of repetitive DNA sequences, including satellites, minisatellites, microsatellites and transposable elements. Despite intensive study in recent decades, the molecular forces that generate, propagate and maintain repetitive DNAs in the genome are still under discussion [10]. Possible functions of satellite DNAs have been studied in several groups of animals, evidencing that these sequences may play an important role at the chromosomal and nuclear level [8,9,11-16].

This report presents a comparative study of the *H. malabaricus *karyomorphs belonging to Group I (A, B, C and D) by means of distinct chromosomal markers, including silver-stained nucleolar organizer regions (Ag-NORs) and chromosomal location of repetitive sequences (18S and 5S rDNA, and satellite 5S*Hind*III-DNA), through fluorescence in situ hybridization (FISH), in order to evaluate the evolutionary relationships among them.

## Methods

### Mitotic chromosome preparations

Chromosome preparations were obtained from *H. malabaricus *specimens from different river basins belonging to karyomorphs A, B, C and D, as specified in Table 2. The animals were first injected in the abdominal region with a 0.025% aqueous solution of colchicine at a dose of 1 ml/100 g of weight. After 50–60 minutes, the specimens were sacrificed, and the chromosomal preparations were obtained from cells of the anterior kidney [17].

**Table 2:** Collection sites of *Hoplias malabaricus*, with the respective karyomorphs and sample sizes.

**Locality**	**Karyomorph**	**N**
Descalvado (SP) – Pântano River	**A**	8 – Male 6 – Female
Parque Florestal do Rio Doce (MG) – lagoons: Doce River	**B**	5 – Male 6 – Female
Poconé (MT) – lagoons: Bento Gomes River	**C**	5 – Male 8 – Female
São Carlos (SP) – UFSCar reservoir: Monjolinho Stream	**D**	10 – Male 7 – Female

SP = São Paulo, MT = Mato Grosso, and MG = Minas Gerais Brazilian States.

### Probes

Three tandem-arrayed DNA sequences isolated from the *H. malabaricus *genome were used. The first probe contained a 5S rDNA repeat copy and included 120 base pairs (bp) of the 5S rRNA encoding gene and 200 bp of the non-transcribed spacer (NTS) [3]. The second probe contained a copy of the repetitive satellite 5S*Hind*III-DNA sequence with 360 bp composed of a 95-bp segment with similarity to the 5S rRNA gene of the first probe and a 265-bp segment similar to the NTS of the first probe [3]. The third probe corresponded to a 1,400 bp-segment of the 18S rRNA gene obtained via PCR from nuclear DNA [5].

### FISH procedure, sequential Ag-NOR detection and karyotype analysis

Fluorescence *in situ *hybridization (FISH) was performed on mitotic chromosome spreads [18]. The probes were labeled by nick translation with biotin-14-dATP (Bionick labeling system-Invitrogen). The metaphase chromosome slides were incubated with RNAse (40 μg/ml) for 1.5 h at 37°C. After denaturation of chromosomal DNA in 70% formamide/2× SSC at 70°C, spreads were incubated in 2× SSC for 4 min at 70°C. Hybridization mixtures containing 100 ng of denatured probe, 10 mg/ml dextran sulfate, 2× SSC, and 50% formamide in a final volume of 30 μl were dropped on the slides, and the hybridization was performed overnight at 37°C in a 2× SSC moist chamber. These hybridization conditions were previously described for the 5S rDNA and 5S*Hind*III-DNA probes in order to avoid possible cross-hybridization [3] Post-hybridization washes were carried out at 37°C in 2× SSC, 50% formamide for 15 min, followed by a second wash in 2× SSC for 15 min, and a final wash at room temperature in 4× SSC for 15 min. Detection of hybridized probes was carried out with 0.07% avidin-FITC conjugate (Sigma) in C buffer (0.1 M NaHCO_3_, 0.15 M NaCl) for 1 h followed by 2 rounds of signal amplification using 2.5% anti-avidin biotin conjugate (Sigma) in blocking buffer (1.26% NaHCO_3_, 0.018% sodium citrate, 0.0386% triton and 1% non-fat dried milk) for 30 min. Each treatment with anti-avidin biotin conjugate was followed by a treatment with avidin-FITC. The treatments with avidin-FITC and anti-avidin-biotin were conducted in a 2× SSC moist chamber at 37°C. After each amplification step, the slides were washed 3 times for 5 min each in blocking buffer at 42°C. The post-hybridization washes were performed on a shaker (150 rpm). The chromosomes were counterstained with propidium iodide (50 μg/mL) and analyzed with an Olympus BX50 epifluorescence microscope. The chromosome images were captured using CoolSNAP-Pro software (Media Cybernetic). Slides previously treated by FISH were washed with water and dehydrated with washes in 75%, 85% and 100% ethanol for 5 minutes for each concentration. After air drying, the slides were prepared for the Ag-NORs detection [19]. Approximately 30 metaphase spreads were analyzed per specimen to determine the diploid chromosome number and karyotype structure. The chromosomes were classified as metacentric (m), submetacentric (sm) or subtelocentric (st), according to arm ratios [20].

## Results

**Figure 1:**
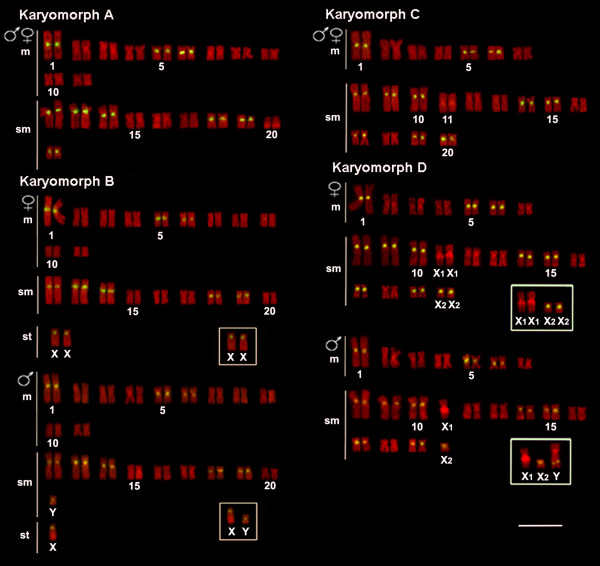
**Karyotypes of *Hoplias malabaricus *(karyomorphs A-D) arranged from chromosomes probed with 5S*Hind*III-DNA satellite sequences (yellow signals) and counterstained with propidium iodide**. The sex chromosomes of karyomorphs B and D are boxed. Bar = 5 μm.

**Figure 2:**
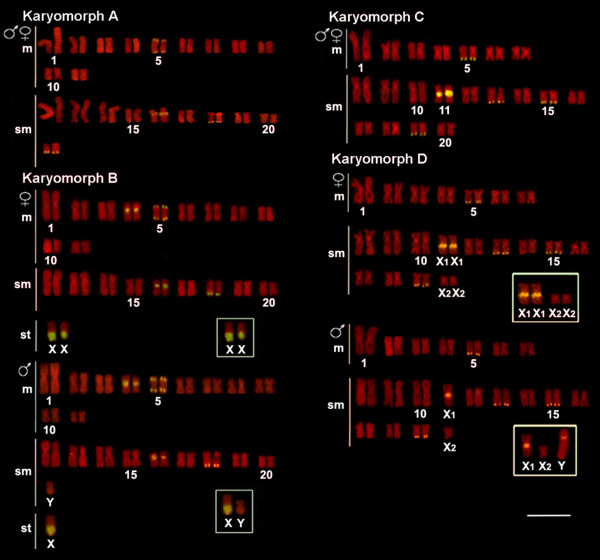
**Karyotypes of *Hoplias malabaricus *(karyomorphs A-D) arranged from chromosomes probed with 18S rDNA (yellow signals) and counterstained with propidium iodide**. The sex chromosomes of karyomorphs B and D are boxed. Bar = 5 μm.

**Figure 3:**
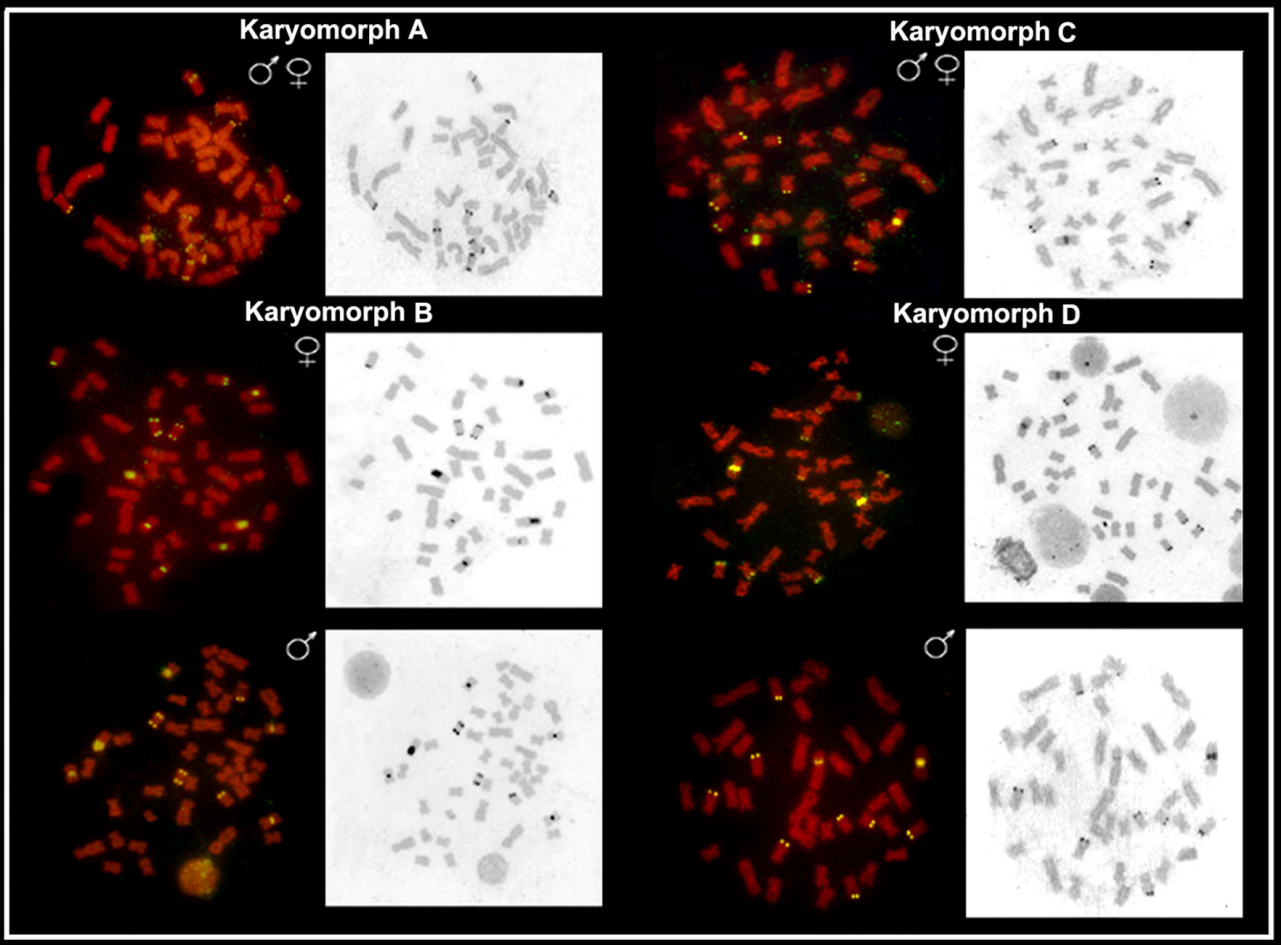
***Hoplias malabaricus *FISH metaphases (karyomorphs A-D), showing the 18S rDNA and Ag-NORs sites with sequential analysis**. Note the general correspondence between the number and location of the 18S rDNA cistrons and Ag-NORs.

**Figure 4:**
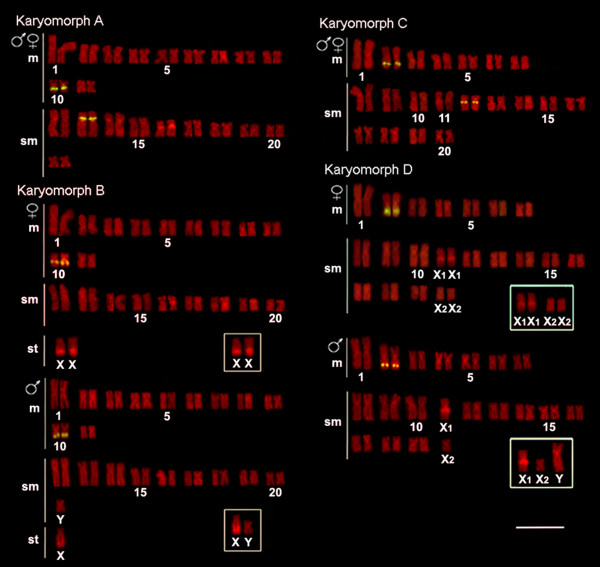
**Karyotypes of *Hoplias malabaricus *(karyomorphs A-D) arranged from chromosomes probed with 5S rDNA (yellow signals) and counterstained with propidium iodide**. The sex chromosomes of karyomorphs B and D are boxed. Bar = 5 μm.

**Figure 5:**
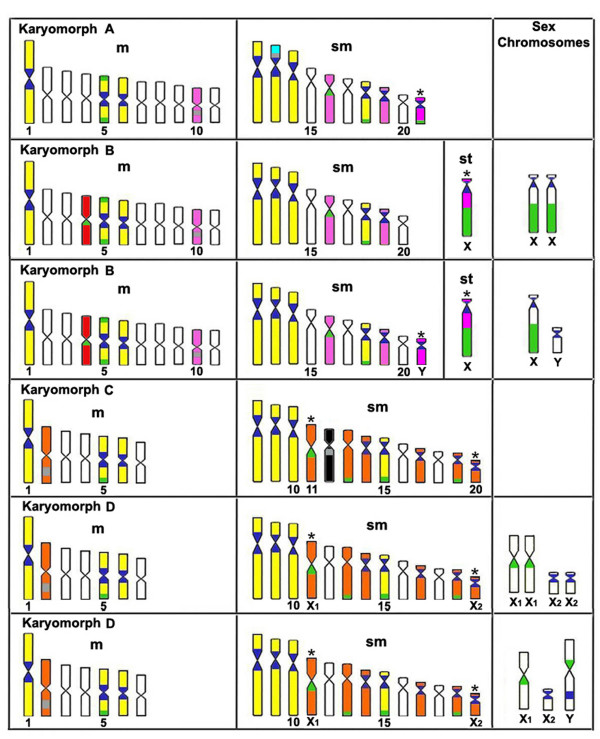
**Representative idiogram of *Hoplias malabaricus *karyomorphs A-D, on the basis of repetitive DNA sequences analyzed**. The sites location of the satellite 5S*Hind*III-DNA, 18S rDNA and 5S rDNA on the chromosomes is indicated in deep blue, green and gray, respectively. Yellow indicates the corresponding chromosomes of karyomorphs A-B-C-D; pink indicates the corresponding chromosomes of karyomorphs A-B and in orange are indicated the corresponding chromosomes of karyomorphs C-D. The chromosomes bearing markers that are exclusive of karyomorphs A, B and C are indicated in blue, red and black, respectively. The asterisks indicate probable relationships between the sex chromosomes of karyomorphs B and D with some autosome pairs of karyomorphs A and C, respectively.

Sites of 5S*Hind*III-DNA, 5S rDNA and 18S rDNA were clearly detected by the FISH procedures, allowing their clear identification and location in the chromosomes of *H. malabaricus *(Figures 1, 2, 3 and 4). These data were organized in the form of idiograms (Figure 5) to facilitate the comparative analysis between the karyomorphs.

The satellite 5S*Hind*III-DNA was mapped in the centromeric region of several chromosome pairs. The karyomorphs A and B presented eight chromosome pairs (nos. 1, 5, 6, 12, 13, 14, 18 and 19) carrying these sites. Additionally, 5S*Hind*III-DNA sites were also highlighted on chromosome pair 21 of karyomorph A and on chromosomes X and Y of karyomorph B. On the other hand, the karyomorphs C and D presented 10 chromosome pairs (nos. 1, 5, 6, 8, 9, 10, 14, 15, 17 and 19), in addition to sites located on chromosomes no. 20 of karyomorph C and X_2 _and Y of karyomorph D (Figures 1 and 5).

18S rDNA sites were proximal to the centromere or in the telomeric region of the chromosomes. In the latter case, bitelomeric sites, i.e., present in both telomeric regions, could also be seen. Karyomorphs A and B presented four chromosome pairs bearing such sites, three of them (nos. 5, 16 and 18) showing a conserved location in both karyomorphs. The fourth site was exclusive to karyomorph A (pair no. 21) or karyomorph B (pair no. 4). In addition, a conspicuous cistron was present on the X chromosome of karyomorph B, occupying a great extent of its long arms. In karyomorphs C and D, five chromosome pairs (nos. 5, 11, 13, 15 and 19) carried 18S rDNA sites. The chromosome 11 of karyomorph C showed correspondence with chromosome X_1 _of karyomorph D, both in the form, size and location of a conspicuous NOR site on the long arms, proximal to the centromere. In turn, the Y chromosome of karyomorph D also showed a proximal 18S rDNA site in the short arms (Figures 2, 3 and 5). In general, there was perfect correspondence between the number and location of 18S rDNA and Ag-NOR sites (Figure 3).

Cytogenetic mapping of the 5S rDNA sequences showed conserved markers only in corresponding chromosomes of karyomorphs A-B (metacentric pair no. 10) or karyomorphs C and D (metacentric pair no. 2), both with interstitial sites on the long arms. However, an exclusive proximal cluster was located in the short arms of the submetacentric pair no. 13 of karyomorph A and in the long arms of the submetacentric pair no. 12 of karyomorph C (Figures 4 and 5).

**Figure 6:**
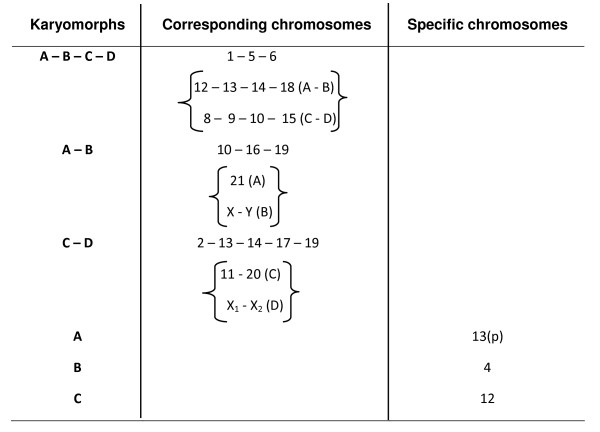
**Chromosomal pairs bearing repetitive DNA sites with correlation or specificity for *Hoplias malabaricus *karyomorphs**. The chromosomes between curly brackets are indicated according their position in the karyotypes.

Figure 6 summarizes all of the corresponding chromosomes of karyomorphs A-D, karyomorphs A-B, or karyomorphs C-D, as well as the chromosomes that showed sites exclusive to each karyomorph, considering the repetitive DNAs analyzed.

## Discussion

Despite the differences regarding the diploid number and the occurrence of differentiated sex chromosomes, the four karyomorphs possess a relatively homogeneous karyotypic structure, basically formed by meta-submetacentric chromosomes, constituting an apparently related evolutionary group – Group I – in *H. malabaricus *[1]. Karyomorphs A and B have 2n = 42 chromosomes, suggesting that karyomorph B was most likely derived from the emergence of a sex chromosome system XX/XY, where X corresponds to the only subtelocentric chromosome of the karyotype. Such a relationship also seems to be applicable for the karyomorphs C (2n = 40) and D (2n = 39 males/2n = 40 females), in that the latter could also have been derived by the emergence of a multiple sex chromosomes system, X_1_X_1_X_2_X_2_/X_1_X_2_Y [1].

The cytogenetic mapping of different repetitive DNA sequences provided reliable chromosomal markers, which allowed the determination of relationships among different karyomorphs. The use of these same markers in a comparative analysis among different populations of karyomorph A also demonstrated a continuing genomic differentiation in this group, allowing the detection of recent evolutionary events, independent of great variations in karyotypes [5]. In fact, repetitive DNAs are highly dynamic throughout evolution, allowing their employment in evolutionary studies. Evaluation of all of the obtained markers shows seven corresponding chromosome pairs in the four karyomorphs, four corresponding pairs in karyomorphs A-B and seven corresponding pairs in karyomorphs C-D, along with some exclusive chromosomes (Figure 6). In addition to corroborate the inclusion of karyomorphs A-D in the same major evolutionary group (Group I), the results provide further evidence for a greater proximity between the karyomorphs A-B and karyomorphs C-D, and also identify some of their peculiarities.

The repetitive 5S*Hind*III-DNA sequence is a tandemly organized DNA family that shares similarity with 5S rDNA and probably originated from duplicated segments of this ribosomal DNA class [3]. This satellite DNA family was relatively common in the *H. malabaricus *genome, with 18 chromosomal sites in karyomorphs A-B and 22 sites in karyomorphs C-D. Although 18 sites of 5S*Hind*III-DNA was the common situation found in karyomorph A, a comparative analysis among different populations of this karyomorph showed 22 sites for only one of them. However, such additional sites showed no correspondence with those present in the chromosomes of karyomorphs C and D [5]. All 5S*Hind*III-DNA sites had an exclusive location in the centromeric region of chromosomes, consistent with previous findings for other Erythrinidae populations [4]. It is well known that centromeric regions are rich in repetitive DNAs, as seen in several organisms, including humans, mice, maize, fruit flies and yeast [21]. It is therefore likely that 5S*Hind*III-DNA repetitions may have some structural or functional role in *H. malabaricus *chromosomes as components of their centromeric DNA [3].

In higher eukaryotes, the rRNA genes are organized as two distinct multigene families, represented by 45S rDNA (18S+5.8S+28S) and 5S rDNA. Both families are composed of tandemly repeated units, with hundreds to thousands of copies. Multiple copies of the 45S rDNA correspond to the nucleolar organizing regions [22]. Although NORs are frequently telomeric in fish, they show a great variability in this group, both in position, number and size of the cistrons [7,23]. A comparative analysis between the 18S rDNA sites and the Ag-NORs showed great similarity regarding the location of the nucleolar organizing regions in *H. malabaricus*. Any decreases in the number of Ag-NORs, observed in some karyomorphs, can be attributed to differential gene activity among the 18S rDNA sites in the cells, since the Ag-NORs represent only those cistrons that were active in the preceding interphase [24,25]. Along with some exclusive chromosome markers for each karyomorph, corresponding chromosomes could also be observed, as is the case for metacentric no. 5 (karyomorphs A-D) and the submetacentrics nos. 18 (karyomorphs A and B) and 15 (karyomorphs C and D). The two latter chromosomes, although occupying distinct karyotype positions between karyomorphs, may correspond to the same chromosome, not only by their shape and size, but also by sharing a 5S*Hind*III-DNA site. In turn, in karyomorphs C and D, the metacentric no. 5 harbored a single 18S rDNA site. In karyomorphs A and B this chromosome showed bitelomeric NORs, indicating that additional events had occurred allowing the acquisition of new sites of rDNA (Figure 5). It is interesting to note that the presence of bitelomeric NORs in *H. malabaricus *is relatively frequent in karyomorphs A and B [6,26-28].

When compared to 18S rDNA and 5S*Hind*III-DNA, the 5S rDNA was a more specific marker, since corresponding chromosomes were not found among the four karyomorphs, in addition to some exclusive sites in karyomorphs A and C. In this way, it seems that karyotype differentiation in Group I did not retain any basal characteristic concerning this chromosomal marker. In this context, the 5S rDNA appears to have gone through karyotypic changes more pronounced than the 18S rDNA and 5S*Hind*III-DNA, since only evolutionary more related karyomorphs, i.e., A-B and C-D, show corresponding chromosomes. Coincidentally, sites of 5S rDNA were shown to be good population markers in *H. malabaricus*, because they showed significant differences even among populations from the same karyomorph [5].

5S rRNA genes are generally found in an interstitial position on chromosomes from the majority of fish species [29], as well as in other vertebrates [30-33], suggesting that such a pattern of distribution is not a coincidence. Furthermore, its chromosomal location is usually not syntenic with the 45S rDNA sites. Although the populations analyzed here show that the location of 18S and 5S rDNA sites were always independent, synteny was already found in a population of karyomorph A [5], highlighting again the dynamic behavior of rDNAs throughout the karyotype evolutionary process of *H. malabaricus*. Telomeric regions would be more conducive to genetic material transfer between chromosomes due their proximity inside the interphase nucleus [34]. This fact could be associated with the greater numerical conservation of the 5S rDNA sites in relation to 18S rDNA sites in fish, possibly due to a preferential location in interstitial and telomeric regions of the chromosomes, respectively [29].

Concerning the sex chromosome systems, the distribution of 5S*Hind*III-DNA and 18S rDNA also indicates a likely correlation between some chromosomes of karyomorphs A and C, with the sex chromosomes present in karyomorphs B and D, respectively. This is the case for chromosome no. 21 of karyomorph A, which shares the 18S rDNA and 5S*Hind*III-DNA sites with chromosomes X and Y of karyomorph B, in addition to showing a marked similarity in relation to the size and morphology of chromosome Y (Figure 5). In the same way, chromosome no. 11 of karyomorph C is similar to chromosome X1 of karyomorph D, in both the physical location of an 18S rDNA site and the correspondence between CMA3 positive regions (data not shown). It is likely that the differentiation of the XX/XY system of karyomorph B has occurred from a heterochromatinization process. This resulted in a large subtelocentric X chromosome that harbors a conspicuous heterochromatic block on the long arms, which co-locates with a NOR site and presents a polymorphic behavior [26]. On the other hand, a clear case of translocation is associated with the origin of the X_1_X_1_X_2_X_2_/X_1_X_2_Y sex chromosome system in karyomorph D, resulting in a large Y chromosome present only in males and a consequent diploid number reduction in this sex [35,36]. The data presented here suggest that the sex chromosomes of karyomorphs B and D were derived from chromosomes 21 and 11 of karyomorphs A and C, respectively. Ongoing studies with additional chromosomal markers will provide a conclusive analysis of the evolution of sex chromosome systems in *H. malabaricus*.

## Conclusion

Repetitive DNAs were important for the genomic evolutionary process of *H. malabaricus*, as evidenced by the presence and distribution of these sequences on chromosomes. These findings lend further support to the idea that Group I is representative of karyomorphs that are closely related and suggest a greater evolutionary proximity between the karyomorphs A-B and karyomorphs C-D, as well as the probable chromosomal origin of the sex systems.

## Authors' contributions

MBC carried out the cytogenetic analyses and drafted the manuscript. CM helped in cytogenetic analysis and drafted the manuscript. LACB designed and coordinated the study, and drafted and revised the manuscript. All authors read and approved the final manuscript.
