# Correction: Comparative chromosome mapping of repetitive sequences. Implications for genomic evolution in the fish, *Hoplias malabaricus*

**DOI:** 10.1186/1471-2156-14-24

**Published:** 2013-04-09

**Authors:** Marcelo B Cioffi, Cesar Martins, Luiz AC Bertollo

**Affiliations:** 1 Departamento de Genética e Evolução, Universidade Federal de São Carlos, São Carlos, SP, Brazil; 2 Departamento de Morfologia, UNESP – Universidade Estadual Paulista, Instituto de Biociências, Botucatu, SP, Brazil

## Correction

After the publication of this work [1] the following errors were brought to the authors’ attention: Figure two contained a mistake that occurred during the editing process and karyotype assemblage so that two chromosome pairs (X_1_ and X_2_) were inadvertently duplicated in the karyotype of the female specimen - karyomorph D. The correct figure is given below (Figure 1).

**Figure 1:**
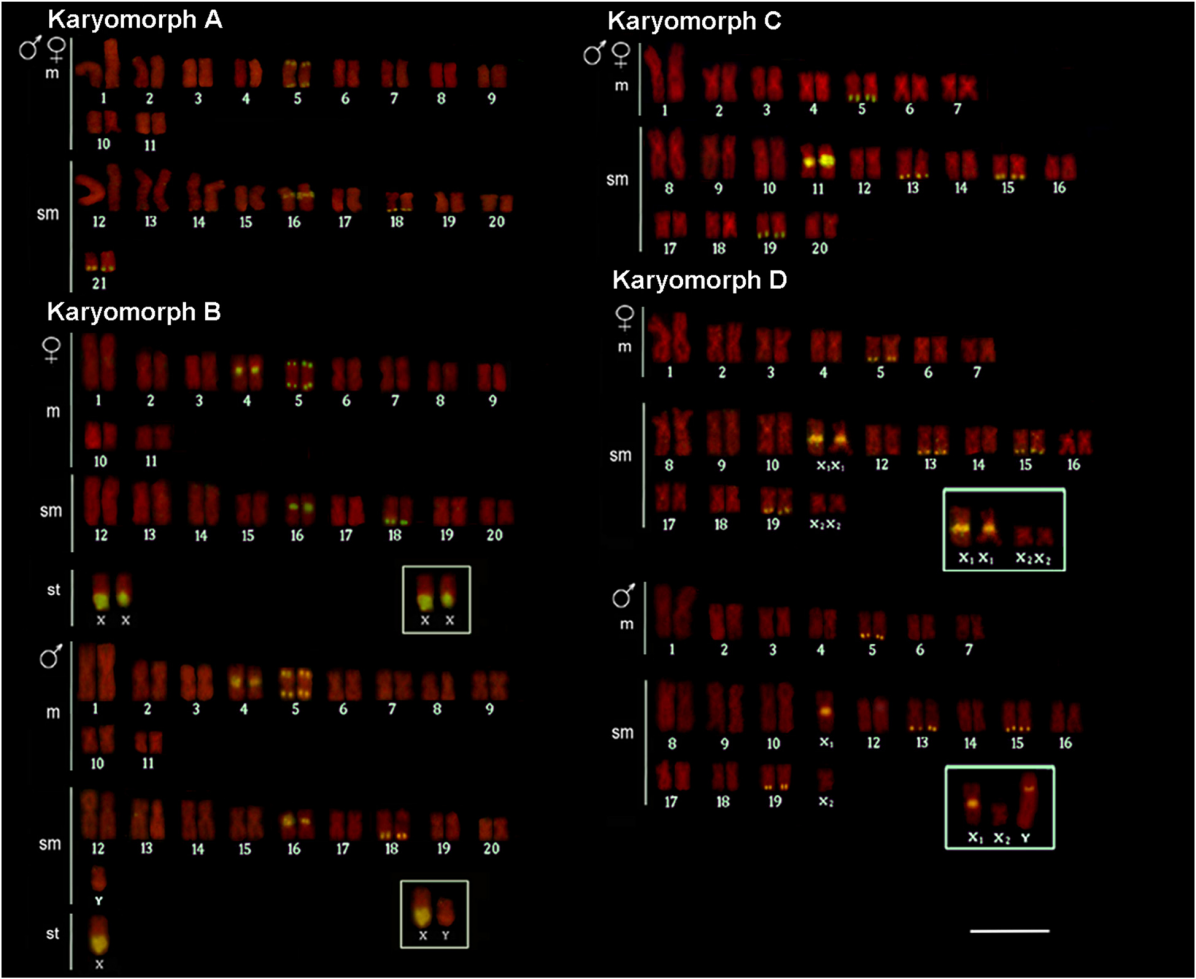
**Karyotypes of *Hoplias malabaricus *(karyomorphs A-D) arranged from chromosomes probed with 18S rDNA (yellow signals) and counterstained with propidium iodide.** The sex chromosomes of karyomorphs B and D are boxed. Bar = 5 μm.

The authors would like to apologise for any confusion caused by this mistake.
